# Genomic-Bioinformatic Analysis of Transcripts Enriched in the Third-Stage Larva of the Parasitic Nematode *Ascaris suum*

**DOI:** 10.1371/journal.pntd.0000246

**Published:** 2008-06-18

**Authors:** Cui-Qin Huang, Robin B. Gasser, Cinzia Cantacessi, Alasdair J. Nisbet, Weiwei Zhong, Paul W. Sternberg, Alex Loukas, Jason Mulvenna, Rui-Qing Lin, Ning Chen, Xing-Quan Zhu

**Affiliations:** 1 Laboratory of Parasitology, College of Veterinary Medicine, South China Agricultural University, Guangzhou, Guangdong Province, People's Republic of China; 2 College of Life Sciences, Longyan University, Fujian Province, People's Republic of China; 3 Department of Veterinary Science, The University of Melbourne, Werribee, Victoria, Australia; 4 Parasitology Division, Moredun Research Institute, Penicuik, United Kingdom; 5 Biology Division, California Institute of Technology, Pasadena, California, United States of America; 6 Helminth Biology Laboratory, Division of Infectious Diseases and Immunology, Queensland Institute of Medical Research, Brisbane, Queensland, Australia; Yale Child Health Research Center, United States of America

## Abstract

Differential transcription in *Ascaris suum* was investigated using a genomic-bioinformatic approach. A cDNA archive enriched for molecules in the infective third-stage larva (L3) of *A. suum* was constructed by suppressive-subtractive hybridization (SSH), and a subset of cDNAs from 3075 clones subjected to microarray analysis using cDNA probes derived from RNA from different developmental stages of *A. suum*. The cDNAs (n = 498) shown by microarray analysis to be enriched in the L3 were sequenced and subjected to bioinformatic analyses using a semi-automated pipeline (ESTExplorer). Using gene ontology (GO), 235 of these molecules were assigned to ‘biological process’ (n = 68), ‘cellular component’ (n = 50), or ‘molecular function’ (n = 117). Of the 91 clusters assembled, 56 molecules (61.5%) had homologues/orthologues in the free-living nematodes *Caenorhabditis elegans* and *C. briggsae* and/or other organisms, whereas 35 (38.5%) had no significant similarity to any sequences available in current gene databases. Transcripts encoding protein kinases, protein phosphatases (and their precursors), and enolases were abundantly represented in the L3 of *A. suum*, as were molecules involved in cellular processes, such as ubiquitination and proteasome function, gene transcription, protein–protein interactions, and function. *In silico* analyses inferred the *C. elegans* orthologues/homologues (n = 50) to be involved in apoptosis and insulin signaling (2%), ATP synthesis (2%), carbon metabolism (6%), fatty acid biosynthesis (2%), gap junction (2%), glucose metabolism (6%), or porphyrin metabolism (2%), although 34 (68%) of them could not be mapped to a specific metabolic pathway. Small numbers of these 50 molecules were predicted to be secreted (10%), anchored (2%), and/or transmembrane (12%) proteins. Functionally, 17 (34%) of them were predicted to be associated with (non-wild-type) RNAi phenotypes in *C. elegans*, the majority being embryonic lethality (Emb) (13 types; 58.8%), larval arrest (Lva) (23.5%) and larval lethality (Lvl) (47%). A genetic interaction network was predicted for these 17 *C. elegans* orthologues, revealing highly significant interactions for nine molecules associated with embryonic and larval development (66.9%), information storage and processing (5.1%), cellular processing and signaling (15.2%), metabolism (6.1%), and unknown function (6.7%). The potential roles of these molecules in development are discussed in relation to the known roles of their homologues/orthologues in *C. elegans* and some other nematodes. The results of the present study provide a basis for future functional genomic studies to elucidate molecular aspects governing larval developmental processes in *A. suum* and/or the transition to parasitism.

## Author Summary

In the present study, we constructed a cDNA library enriched for molecules of the infective third-stage larva (L3) of *Ascaris suum*, the common roundworm of pigs. Using the method of suppressive-subtractive hybridization (SSH), we explored transcription of a subset of molecules by microarray analysis and conducted bioinformatic analyses to characterize these molecules, map them to biochemical pathways, and predict genetic interactions based on comparisons with *Caenorhabditis elegans* and/or other organisms. The results provide interesting insights into early molecular processes in *A. suum*. Approximately 60% of the L3-enriched molecules discovered had homologues in *C. elegans*. Probabilistic analyses suggested that a complex genetic network regulates or controls larval growth and development in *A. suum* L3s, some of which might be involved in or regulate the switch from the free-living to the parasitic stage. Functional studies of these molecules to elucidate developmental processes in *Ascaris* could assist in identifying new targets for intervention.

## Introduction

Parasitic nematodes are of major socio-economic importance in animals. For example, hundreds of millions of people are infected with geohelminths (soil-transmitted worms), such as blood-feeding hookworms *Ancylostoma duodenale* and/or *Necator americanus*, *Trichuris trichiura* and *Ascaris* spp. [1], causing serious adverse effects on human health, particularly in children. Similarly, parasitic nematodes of livestock, such as pigs, also cause substantial economic losses due to subclinical and clinical diseases, with billions of dollars spent annually on the treatment and control of gastro-intestinal nematodes. In addition to the socioeconomic impact that these parasites have, there is potential for the emergence of resistance in them against all of the main classes of (nematocidal) compounds used to treat the diseases they cause [2]–[5]. Therefore, there is a significant need to work toward discovering new compounds to control these parasites. Gaining an improved understanding of the molecular basis of parasite development provides such an avenue.

Compared with the free-living nematode *Caenorhabditis elegans*, there is very little information on fundamental molecular aspects of development in parasitic nematodes [6]–[8]. Since the genome sequence of *C. elegans* was published in 1998 [9], many aspects of the molecular biology of this nematode have been elucidated. For instance, microarray analyses have been used to examine developmental and gender-enriched gene expression [10],[11], and the functions of more than 96% of the *C. elegans* genes have been assessed by double-stranded RNA interference (RNAi, or gene silencing; [12]) [13]–[18]. Comparative analyses of genetic data sets have shown that parasitic nematodes usually share ∼50–70% of genes with *C. elegans* (e.g., [19],[20]). There is similarity in other features (such as basic body plan and moulting) between *C. elegans* and parasitic nematodes, suggesting that some molecular pathways are relatively conserved [8],[21]. Understanding the pathways linked to basic nematode biology and development could have important implications for finding new ways of disrupting these pathways and thus facilitate the identification of new drug targets.

Despite the advances in genomic technologies [7], [22]–[29] and the study of *C. elegans*, there is a paucity of information on the genomics of parasitic nematodes of animals, particularly in relation to development. Also considering the major socioeconomic impact of *Ascaris* and ascariasis in humans and pigs [30]–[32], several characteristics, including the large size of the adult worm (providing the opportunity of investigating individual organ systems and tissues), the ability to maintain *Ascaris* in the pig, store eggs and culture larvae *in vitro* for relatively long periods of time (months to years) [32] as well as the discovery that RNAi achieves “cross-species” gene silencing for a selected number of genes [33],[34] and the imminent genome sequence (http://www.sanger.ac.uk/Projects/Helminths/) all indicate that *Ascaris* could serve as a powerful model system for investigating reproductive and developmental processes in nematodes.

In the present study, *Ascaris* from pigs was used to study molecules abundantly transcribed in the infective third-stage larva (L3). Following the oral ingestion of *Ascaris* eggs by the host (human or pig), L3s are released and then invade/penetrate predominantly the caecal wall [35] to then undergo hepato-pulmonary migration, after which ultimately the adult females and males establish and develop in the small intestine [36],[37]. The molecular mechanisms linked to host invasion and parasite development are largely unknown. Here, we constructed an L3-enriched cDNA library using the method of suppressive-subtractive hybridization (SSH), explored transcription of a representative subset of molecules by microarray analysis and conducted bioinformatic analyses to characterize these molecules, map them to biochemical pathways and predict genetic interactions based on comparisons with *C. elegans* and/or other organisms.

## Materials and Methods

### Production of Different Developmental Stages of *Ascaris*

Experimental pigs (8–12 weeks of age) were purchased from and maintained in the Experimental Animal Center of South China Agricultural University. These pigs were treated humanely, according to the Animal Ethics procedures and guidelines of the People's Republic of China. Adult worms (males and females) of *A. suum* were collected from the small intestines of pigs from an abattoir in Shenzhen, China. Infective eggs and infective L3s of *A. suum* were produced according to the methods described previously [38]. In brief, eggs from the uteri of adult females of *A. suum* were collected and incubated at 28°C for 28 days to allow them to develop to infective eggs (containing infective L3s). To obtain pure infective L3s, 7.5% v/v sodium hypochlorite was used to treat the larvated eggs at 37°C overnight and then the eggs were shaken with glass-beads; then, the exsheathed L3s and shells were separated by density gradient centrifugation using lymphocyte separating medium (LSM) [38]. Following the experimental infection of helminth-free pigs with infective *Ascaris* eggs as described previously [39], the L3s from livers and in lungs as well as L4s in intestines were isolated according to an established method [40]. All parasite materials were snap-frozen in liquid nitrogen prior to storage at −70°C.

### Construction of the cDNA Library by Subtractive-Suppressive Hybridization (SSH)

Total RNA was isolated from adult females and males, different larval stages or eggs of *A. suum* using TriPure reagent (Roche) as recommended by the manufacturer. Equal amounts of total RNA from each stage or sex were pooled. The mRNA was isolated using the Oligotex mRNA Kit (Qiagen), following the manufacturer's protocol. SSH was carried out using the PCR-Select cDNA Subtraction kit (Clontech), according to the manufacturer's protocol. In brief, cDNA synthesized from mRNAs from infective L3s was subtracted against cDNA synthesized from the pooled mRNA from all other stages included herein. The SSH library was constructed using infective L3s as the tester and pooled cDNAs from all other stages as the driver. The effectiveness of this subtraction process has already been demonstrated in previous studies [41],[42]. The cDNA obtained following SSH was cloned into the pGEM-T Easy plasmid vector (Promega) and competent *Escherichia coli* (JM109) transformed. Positive clones, picked randomly (based on blue/white selection), were grown overnight in Luria Bertani (LB) medium (shaking, 37°C). Individual inserts were PCR-amplified using “nested primers” 1 and 2R from the Subtraction kit (Clontech) and examined by agarose electrophoresis.

### Preparation of Microarray Slides

Clones (n = 3075) from the subtracted library were picked and cultured overnight in LB containing ampicillin (1000 IU/ml) in sealed 96-well blocks. Five µl of culture suspension from each well were transferred into individual wells thermocycling (96-well) plates and the inserts PCR-amplified using primers 1 and 2R. Following a 10 min denaturation step at 94°C, the amplification proceeded for 25 cycles of 10 s at 94°C, 30 s at 68°C and 1.5 min at 72°C, with a final extension for 5 min at 72°C. Products were resolved in agarose gels, ethanol precipitated, re-suspended in 16 µl of “spotting solution” (Shanghai BioStar Genechip, Inc) to a final concentration of ∼500 ng per µl, before being printed on to glass slides (in duplicate) using a robotic arrayer. Sixteen blanks (using spotting solution only) and the same number of negative (irrelevant cDNAs with no relationship to *Ascaris*) were also printed on to slides and served as negative controls; β-actin of *A. suum* served as a positive control to assess the efficiency of labeling and hybridization. The slides were air-dried for 2 h, and cDNA in the spots were cross-linked at 254 mJ. The printed slides were stored at 4°C.

### Labeling of cDNA Probes with Fluorescent Dyes, and Microarray Analysis

The cDNAs produced from total RNA from *A. suum* eggs, infective L3s, L3s isolated from pig liver or lung, fourth-stage larvae (L4s), adult males or females [as described in the section ‘Construction of the cDNA Library by Subtractive-Suppressive Hybridization (SSH)’] were labeled with cyanine dyes. Cy3 or Cy5-dCTP was incorporated into cDNA produced from 30 µg of total RNA by direct labeling in a reverse transcription reaction using an oligo (dT) primer. Labeled cDNA was purified using DyeEx columns (Qiagen).

Microarray slides were incubated with a pre-hybridization solution [5×SSC, 1% bovine serum albumin (BSA), 0.1% sodium dodecyl-sulphate (SDS)] for 6 h at 42°C. After pre-hybridization, the microarray slides were incubated with ‘pooled’ Cy3 and Cy5-labeled probes in hybridization solution (5×SSC, 1% BSA, 0.1% SDS), in the dark at 42°C for 18 h, and then washed in solution I (1×SSC, 0.2% SDS) for 10 min, followed by solution II (0.1×SSC, 0.2% SDS) for 10 min at 60°C, according to the protocols provided by Shanghai BioStar Genechip, Inc. A “dye flip” was carried out to control for any bias in hybridization signal between the Cy-labeled cDNA probes (produced for two distinct mRNA populations). The slides were dried and scanned (ScanArray 4000 scanner) using image acquisition software (Shanghai BioStar Genechip Inc.) and a range of laser power and photo-multiplier tube intensities. The mean hybridization signal (derived from four replicates of the same array) were corrected for background, normalized [43], log_2_-transformed and then subjected to statistical analysis employing the students *t*-test in a spreadsheet (Excel, Microsoft, USA). The microarray data were analysed for differential cDNA hybridization (>2.0-fold to 114.3-fold) between L3 and each of the other stages (eggs, lung and liver L3s, L4, adult female and adult male).

### Verification of Differential Hybridization by Reverse Transcription-Coupled Polymerase Chain Reaction (RT-PCR) Analysis

For a subset (n = 17) of representative ESTs (rESTs), RT-PCR was used to verify the differential transcription recorded by microarray analysis. Double-stranded cDNA was synthesized from total RNA (separately) from each stage or sex of *A. suum* using reverse transcriptase (Superscript III, Invitrogen). Briefly, 5 µg of total RNA were added to 14 µl of H_2_O and 1 µl of oligo d(T)n = 12–18 primer (0.5 µg/µl), heated to 70 °C for 10 min and chilled on ice. First- and second-strand cDNAs were synthesized via the addition of 4 µl of first-strand cDNA buffer (250 mM Tris-HCl, pH 8.3, 375 mM KCl and 15 mM MgCl_2_), 2 µl of 0.1 M dithiothreitol, and 1 µl of 10 mM of each dNTP, followed by an incubation at 25 °C (10 min), 42 °C (50 min) and 70 °C (15 min). One-tenth of each double-stranded cDNA produced was then used as a template in the PCR. The transcripts were amplified from individual cDNAs by PCR using oligonucleotide primers (sequences available upon request) designed to each EST. The PCR amplification of a portion (209 bp) of the β-actin gene (accession no. BI594141) using forward primer (5′-CTCGAAACAAGAATACGATG-3′) and reverse primer (5′- ACATGTGCCGTTGTATGATG-3′), previously determined to be present in all developmental stages and both sexes of *A. suum* [44], served as a positive control. Samples without template (no-DNA controls) were included in each PCR run. The following cycling conditions were employed: one cycle at 94 °C (5 min), 94 °C (30 s), 60 °C (30 s) and 72 °C (30 s) for 30 cycles, followed by a final extension of 70 °C (7 min). Following the PCR, 5 µl of individual amplicons were resolved in ethidium bromide-stained agarose gels (2%) and then photographed upon transillumination. The relative band intensities were analyzed using UVIsoft Image Acquisition and Analysis software (UVITEC). The specificity and identity of individual amplicons were confirmed by direct sequencing using the same primers (separately) as employed for their amplification.

### Sequencing and Bioinformatics Analyses

Clones from the SSH cDNA library with increased hybridization in microarray analysis to the infective L3 compared with other stages were sequenced using standard technology [45]. The nucleotide sequences have been deposited in the GenBank database under accession numbers ES290984-ES291074. Following the processing of the sequences (i.e., removal of vector sequences, quality assurance and clustering), contigs or singletons from individual clusters were subjected to BLASTx (NCBI: www.ncbi.nlm.nih.gov) and BLASTn (EMBL-EBI Parasite Genome Blast Server: www.ebi.ac.uk) analysis to identify putative homologues in *C. elegans*, other nematodes and other organisms (e-value of ≤1e-05). Peptides inferred from ESTs were classified functionally using Interproscan (available at http://www.ebi.ac.uk/InterProScan/) employing the default search parameters. WormBase (www.wormbase.org) was interrogated extensively for relevant information on *C. elegans* homologues/orthologues, including RNAi phenotypic, transcriptomic, proteomic and interactomic data. ESTs with homologues/orthologues in *C. elegans* and other nematodes were also subjected to analysis employing the KEGG Orthology-Based Annotation System (KOBAS) (www.kobas.cbi.pku.edu.cn), which predicts the biochemical pathways in which molecules are involved. The open reading frames (ORFs) inferred from selected ESTs with orthologues in *C. elegans* were also subjected to “secretome analysis” using the program SignalP v.2.0 www.cbs.dtu.dk/services/SignalP/), employing both the neural network and hidden Markov models to predict signal peptides and/or anchors [46]–[48]. Also, transmembrane domains were predicted using the program TMHMM (www.cbs.dtu.dk/services/TMHMM/; [49]–[51]), and subcellular localization inferred employing the program WoLF PSORT (http://wolfpsort.org/; [52]).

The method established by Zhong and Sternberg [53] was used to predict the interactions for *C. elegans* orthologues of the L3-enriched molecules from *Ascaris*. In brief, interaction, phenotypic, expression and gene ontology data from fruitfly, yeast, mouse and human were integrated using a naïve Bayesian model to predict genetic interactions among *C. elegans* genes ([45],[53]; Zhong and Sternberg, unpublished). The predicted networks resulting from the analyses were saved in a graphic display file (gdf) format and examined using the graph exploration system available at http://graphexploration.cond.org/. Images were labeled and saved in the joint photographic experts group (jpeg) format.

## Results

To identify molecules transcribed abundantly in the L3 of *A. suum*, an enriched cDNA library was constructed by SSH. From a total of 3075 clones from this library, 2921 (95%) were shown to contain an insert (which could be amplified by PCR). From 2671 (92%) of these clones, amplicons representing single bands of ∼400 to 600 bp in size were produced. These latter amplicons were arrayed (in duplicate) on to slides and then hybridized with Cy3-labeled L3-cDNA or with Cy5-labeled cDNA from eggs, liver/lung L3s, L4s, adult female or adult male of *Ascaris*. Dye flip was conducted to verify the hybridization data. Of the 2671 (duplicate) spots, 1526 had a significant difference in hybridization between infective L3 cDNA and cDNAs from all other stages or sexes of *A. suum*, of which 515 had a >2.0-fold increased hybridization for the L3.

In order to independently verify the hybridization results in the microarray, a PCR-based analysis of a selected subset (n = 17) clones was conducted using specific primer pairs. Having verified the specificity and identity of individual amplicons by sequencing, PCR results were reproducible (based on multiple runs on different days) and ∼94% (16 of 17) concordant with those of the microarray analysis (not shown). There was complete concordance for representative clones associated with a differential signal of ≥3.0-fold in the microarray.

The clones linked to the 515 spots representing increased transcription (>2.0-fold) in infective L3 compared with the other developmental stages or sexes included were subjected to sequencing. The 498 sequences (length: 550±115 bp) determined were then subjected to detailed bioinformatic analyses. There were 91 unique clusters (accession numbers ES290984-ES291074), of which 55 were singletons (sequences determined once). Of 56 molecules (61.5%) with significant similarity to sequences other than *A. suum* in the databases interrogated, 50 (54.9%) had *C. elegans* or *C. briggsae* homologues, and six had similarity to ESTs already sequenced from ascaridoid and/or other parasitic nematodes, and/or other organisms (Table 1). A significant proportion (38.4%) did not have any similarity in sequence to any organisms for which data are presently available. Comparative analysis specifically against *A. suum* EST data sets (n∼42,000) available in public databases confirmed independently that the majority of molecules (>60%) were present exclusively in the infective L3 stage or were orphans.

**Table 1:** Analysis using the Basic Local Alignment Search Tool (BLAST).

Classification of molecules based on similarity (BLASTx) to molecules in	Numbers^a^
*C. elegans* only	16 (6)
*C. elegans* and other ascaridoid nematodes	2
Exclusively other ascaridoid nematodes	1
*C. elegans* and other nematodes	16 (3)
*C. elegans* and other organisms	27 (9)
Exclusively other organisms	5
No known homologue in any organisms	35
Totals	91

Summary of results from bioinformatic and microarray analyses of expressed sequence tags (ESTs) with significant differential transcription between infective third-stage larva (L3) of Ascaris suum and other developmental stages.

a Numbers in brackets refer to *C. elegans* homologues with known non-wildtype RNAi phenotypes (see Table 2).

**Figure 1:**
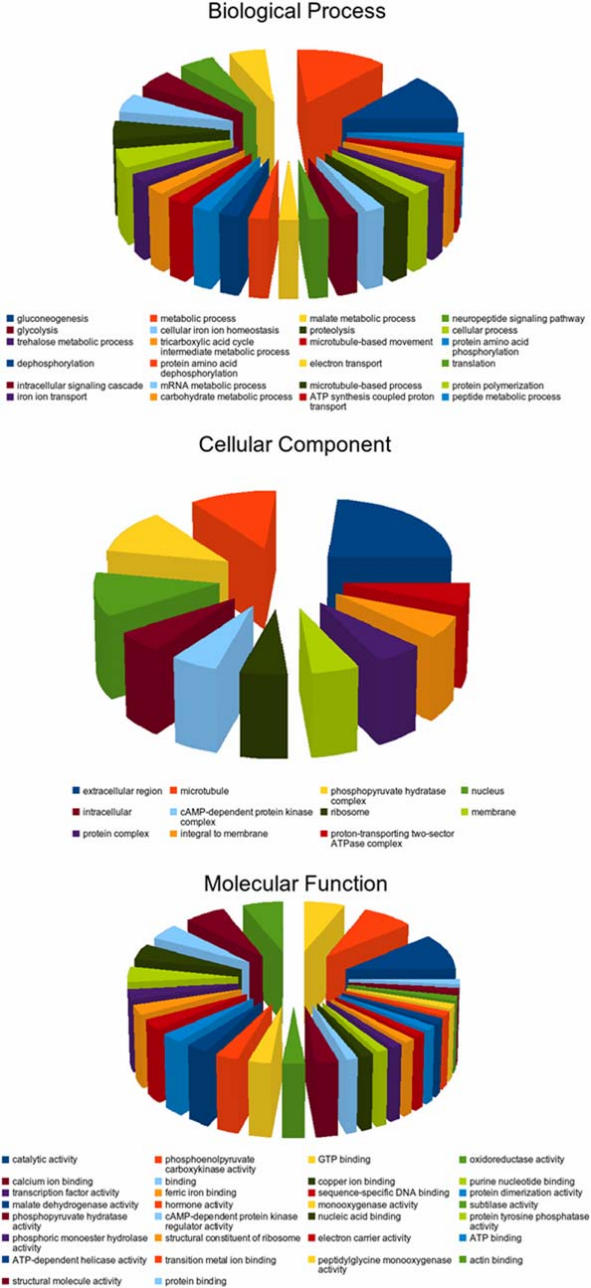
Gene ontologies. Summary of predicted functions and locations for gene products inferred from 32 clusters of molecules enriched in the infective third-stage larva (L3) of *Ascaris suum*, classified according to the gene ontology (GO) categories ‘cellular component’ ‘biological process’ and ‘molecular function’ as well as subcategories within.

As gene ontology (GO) provides a hierarchy that unifies the descriptions of biological, cellular and molecular functions [54], this approach was employed to predict the classification and gene function of molecules enriched in infective L3 of *A. suum*. A summary of the GO categories of these molecules is displayed in Fig. 1. Of the 91 contigs, 32 (35%) could be functionally assigned to ‘biological process’ (n = 38), ‘cellular component’ (n = 17) and ‘molecular function’ (n = 64). The most common subcategories were gluconeogenesis (13%) and metabolic process (13%) within ‘biological process’, extracellular region (24%) within ‘cellular component’, and catalytic activity (11%) and phosphoenolpyruvate carboxykinase activity (8%) within ‘molecular function’ (Table S1).

A focused KOBAS analysis inferred the 50 *C. elegans* orthologues/homologues to be involved in apoptosis and insulin signaling (2%), ATP synthesis (2%), carbon metabolism (6%), fatty acid biosynthesis (2%), gap junction (2%), glucose metabolism (6%) or porphyrin metabolism (2%), although 34 (68%) of them could not be mapped to a specific metabolic pathway (Table 2). Of these 50 molecules, small numbers were predicted to be secreted (10%), anchored (2%) and/or transmembrane (12%) proteins (Table 2). Functionally, 17 (34%) of the 50 molecules were associated with (non-wild-type) RNAi phenotypes in *C. elegans*, the majority displaying embryonic lethality (Emb) (13 types; 58.8%), larval arrest (Lva) (23.5%) and larval lethality (Lvl) (47%) (Table 2).

**Table 2:** *Caenorhabditis elegans* orthologues.

EST code	Size (bp)	*In silico* peptide analysis *	Description of *C. elegans* homologue (Gene code, *gene name*)	RNAi phenotype**	Other ascaridoid nematodes [non-ascaridoid nematodes] ***
Apoptosis; Insulin signalling pathway					
22H01	698	Q/0/Cy	Predicted regulatory subunit of a cAMP-dependent protein kinase (R07E4.6, *kin-2*)	Lva, Lvl, Bmd, Dpy, Unc, Stp	*[Mar, Min, Mja, Ovo, Sst]*
ATP synthesis					
28A02	686	Q/0/Mi	Mitochondrial F1F0-ATP synthase (T05H4.12, *atp-4*)	Emb^1,2^, Ste, Age, Gro	*[Aca, Hco, Ppa, Tci]*
Carbon fixation; Citrate cycle (TCA cycle); Pyruvate metabolism; Reductive carboxylate cycle (CO2 fixation)					
11C02	403	Q/0/Cy	Malate dehydrogenase (F46E10.10)		*Alu [Cbr]*
Fatty acid biosynthesis					
30A01	553	Q/0/Cy	Short-chain dehydrogenase (F09E10.3, *dhs-25*)		*[Hco, Min]*
Gap junction					
13E09	541	Q/0/Cy_Nu	alpha-tubulin (C44B11.3, *mec-12*)	Emb^1,3^, Gro, Lva, Lvl	*[Ace, Bma, Cbr, Hco, Hgl, Mha, Ppa, Tca, Xin]*
Glycolisis/Gluconeogenesis; Phenylalanine, tyrosine and tryptophan biosynthesis					
22H06	460	Q/0/Cy	Enolase (T21B10.2, *enol-1*)	Emb^1^, Lva, Gro, Clr	*Asi [Ace, Aca, Bma, Gpa, Hco, Hgl, Lsi, Mar, Mha, Ovo, Ppa, Ptr, Tsp, Tmu, Wba, Xin]*
15G04	641	Q/0/Mi	Enolase (T21B10.2, *enol-1*)	Emb^1^, Lva, Gro, Clr	*Asi [Ace, Aca, Bma, Gpa, Hgl, Mar, Mha, Ovo, Ppa, Ptr, Tsp, Wba, Xin]*
Glyoxylate and decarboxylate metabolism					
11C02	403	Q/0/Cy	Malate dehydrogenase (F46E10.10)		*Alu [Cbr]*
29E06	469	Q/0/Cy_Nu	Predicted hydrolase, HAD superfamily (F45E1.3)		*[Bma, Hgl, Ppa]*
Huntington's disease					
18C04	829	Q/0/Cy_Nu	Proposed component of the intraflagellar transport (IFT) complex B (F59C6.7, *che-13*)		
Porphyrin metabolism					
2G04	505	Q/0/Pl	Heavy chain ferritin (D1037.3, *ftn-2*)		*[Aca, Ace, Bma, Cbr, Gro, Hco, Hgl, Mha, Mja, Oos, Ppe, Pvu, Ppa, Xin, Zpu ]*
Starch and sucrose metabolism					
18D07	441	S/0/Ex	Putative trehalase (T05A12.2, *tre-2*)		*[Ace, Bma, Cbr, Ovo, ptr, Sst, Sra, Wba]*
Other enzymes					
03C04	520	Q/0/Cy	Peptidylglycine alpha-amidating monooxygenase (Y71G12B.4)	Age	*[Aca, Ace, Bma, Hgl, Mha, Mch, Ovo, Pvu, Tci, Wba]*
21G04	238	S/0/Ex	Peptidylglycine alpha-amidating monooxygenase (Y71G12B.4)	Age	*[Aca, Ace, Bma, Dim, Gro, Min, Mja, Ovo, Wba]*
Unassigned pathway					
01G03	544	Q/0/Nu	Troponin (T22E5.5, *mup-2*)	Gro, Slu, Unc, Stp, Ste	*[Ace, Bma, Dim, Lsi, Min, Ovo, Sst, Tci, Tca, Wba, Xin]*
04G09	542	Q/0/Cy_Nu	Immunoglobin and related proteins (KOG4475, *dim-1*)		*Alu [Aca, Cbr, Hgl, Mja, Mpa, Nam, Pvu, Ppa, Tmu, Wba, Xin]*
07E12	519	S/0/Ex	Unnamed protein (T24D8.5, *nlp-2*)		*[Aca, Hgl, Tca]*
09A01	639	Q/0/Cy_Nu	Subtilisin-like proprotein convertase (C51E3.7, *egl-3*)	Ric, Age	*[Aca, Cbr, Gro, Hgl, Mch, Mha, Min, Mja, Nam, Oos, Ptr, Sst, Tsp, Xin]*
09G10	741	Q/0/Cy	Polyadenylate- binding protein (Y106G6H.2, *pab-1*)	Gro, Stp, transposon silencing abnormal, reproductive system morphology abnormal, Fgc, Pvl, Rup, Bmd, Ste, Emb^1,4^	*[Aca, Ace, Bma, Cbr, Hgl, Mar, Mha, Min, Mja, Mpa, Nam, Ptr, Ppa Ppa, Tci, Tmu]*
10E04	489	Q/0/Ex	Defense-related protein containing SCP domain (F11C7.3, *vap-1*)		*[Tci, Tca]*
13C11	551	A/0/Mi	G protein signaling regulators (C16C2.2, *eat-16*)		*[Mha]*
13E04	472	Q/0/Cy	Unnamed protein (B0546.2)		
18B10	566	Q/1/Ex	Cell differentiation regulator of the Headcase family (K07A1.7)		*[Dim]*
18E12	673	Q/0/Cy	Phosphoenolpyruvate carboxykinase (W05G11.6)		*[Aca, Ace, Cbr, Gro, Hco, Nam, Nbr, Ovo, Tci, Tsp, Tmu, Wba, Xin]*
20D08	506	Q/0/Nu	Protein tyrosine phosphatase (B0244.2, *ida-1*)		*[Min, Ovo, Tca]*
22C06	366	Q/0/Ex	Unnamed protein (F31F6.4, *flp-8*)		*[Aca, Ace, Xin]*
24H07	714	Q/1/Pl	Neural proliferation, differentiation and control protein (C23H4.1, *cab-1*)	Ric	*[Gpa, Gro, Hgl, Xin]*
29F02	471	S/0/Pl	Unnamed protein (H05L03.3)	Fat content increased	*[Aca, Bma, Min, Mja, Mpa, Nam, Ppe, Sst]*
30A01	653	Q/0/Cy	Mitochondrial/plastidial beta-ketoacyl-ACP reductase (F09E10.3, *dhs-25*)		*[Hco, Min]*
30C11	508	Q/0/Cy	Calmodulin and related proteins (EF-Hand superfamily) (C24H10.5, *uvt-2*)		*[Aca, Ace, Bma, Dim, Gro, Hgl, Min, Nam, Xin]*
2G12	653	Q/0/Ex	Alpha crystallins (F52E1.7, *hsp-17*)		*[Dim, Ovo]*
4F10	513	Q/0/Cy	Predicted ATP-dependent RNA heclicase FAL1 (F33D11.10)	Lva, Emb^1,5,6,7,8,9,10,11,12^, early larval arrest, Mul	*[Ace, Bma, Cbr, Gpa, Hco, Hgl, Mar, Nam, Ovo, Pte, Ppa, Sra, Tsp]*
4G11	536	Q/0/Cy	Predicted P-loop ATPase fused to an acetyltransferase (F55A12.8)	Gro, Unc, Pvl, Emb^1^, Lva, Ste, early larval arrest	*[Bma]*
6E01	472	S/0/Ex	Unnamed protein (F56F3.6, *ins-17*)	Sck, Ste, Pvl, transgene subcellular localization abnormal, Emb^1^, Lva	
10D07	639	S/0/Nu	Predicted membrane protein, contains two CBS domains (C52D10.12)		
11F10	566	Q/1/Cy	Cell differentiation regulator of the Headcase family (K07A1.7)		*[Dim]*
12C05	547	Q/0/Cy	Actin regulatory proteins (gelsolin/villin family) (K06A4.3)		*[Cbr, Mch, Mha, Min, Tca]*
13B06	458	Q/1/Pl	Unnamed protein (C44C10.9)		
14B05	259	Q/0/Mi	Uncharacterized conserved protein (F09G8.5)		
18A11	575	Q/0/Mi	Uncharacterized conserved protein (C16C10.11)		*Alu [Aca, Ace, Bma, Hco, Ovo, Ptr, Lsi, Rsi, Wba, Xin]*
18B04	393	Q/0/Ex	Alpha crystallins (F52E1.7, *hsp-17*)		*[Bma, Ovo, Oos]*
18D12	740	Q/2/Pl	Uncharacterized protein, contains CX module (H06I04.6)		*[Mha, Sra]*
24D03	804	Q/0/Ex	Defense-related protein containing SCP domain (F11C7.3, *vap-1*)		*[Tca]*
24F05	412	S/1/Ex	Unnamed protein (Y48D7A.2, *flp-18*)	Emb^1^	
26A11	647	Q/0/Nu	Unnamed protein (ZC8.4, *lfi-1*)		*Alu, Pun [Bma, Cbr, Mha, Min, Ovo, Oos, Srs, Sst, Tca]*
26G12	532	Q/0/Cy	60S ribosomal protein L22 (C27A2.2, *rlp-22*)	Emb^1,13^, , Lva, Ste, Lon, Gro, Pch	*[Tca]*
28A09	455	Q/0/Cy	Phosphoenolpyruvate carboxykinase (W05G11.6)		*[Aca, Ace, Bma, Cbr, Hco, Mch, Min, Ovo, Ppa, Sst, Tci, Tvi, Wba]*
30D01	255	Q/0/Cy	Unnamed protein (F09E10.7)		*[Aca, Ace, Hsc]*

*Ascaris suum* expressed sequence tags (ESTs) (listed according to KOBAS classification) with homologues in *C. elegans* and other nematodes (BLASTn analysis) and with a significantly higher (>2.0-fold) transcription in the infective third-stage larva (L3) compared with other stages. *C. elegans* homologues with known RNAi phenotypes have been also included.

*: Abbreviations used in proteomic analysis: Non-secretory protein (Q), signal anchor (A), secretory protein (S)/ predicted number of transmembrane domains/ predominat cellular location: cytoplasm (Cy); extracellular (Ex); mitochondrial (Mi), nuclear (Nu), plasma membrane (Pl).

**: Abbreviations of RNAi phenotypes (alphabetical): lifespan abnormal (Age), body morphology defect (Bmd), clear (Clr), dumpy (Dpy), embryonic lethal (Emb^1^), general pace of development abnormal early emb (Emb^2^), mitotic spindle abnormal early emb (Emb^3^), embryo osmotic integrity abnormal early emb (Emb^4^), excessive blebbing early emb (Emb^5^), large cytoplasmic granules early emb (Emb^6^), cell cycle slow early emb (Emb^7^), ectopic cleavage furrows early emb (Emb^8^), nuclear reassembly abnormal early emb (Emb^9^), polar body abnormal early emb (Emb^10^), pronuclear size abnormal early emb (Emb^11^), excess maternal pronucleus early emb (Emb^12^), chromosome segregation abnormal karyomeres early emb (Emb^13^), fewer germ cells (Fgc), slow growth (Gro), larval lethal (Let), long body (Lon), larval arrest (Lva), early larva lethal (Lvl), multiple nuclei early emb (Mul), patchy coloration (Pch), protruding vulva (Pvl), aldicarb resistant (Ric), ruptured (Rup), sick (Sck), sluggish (Slu), sterile (Ste), sterile progeny (Stp), uncoordinated (Unc).

***: Abbreviations of nematode species (alphabetical): *Ancylostoma caninum (Aca)*, *Ancylostoma ceylanicum (Ace)*, *Ascaris lumbricoides (Alu)*, *Anisakis simplex (Asi)*, *Brugia malayi (Bma)*, *Caenorhabditis briggsae (Cbr)*, *Dirofilaria immitis (Dim)*, *Globodera pallida (Gpa)*, *Globodera rostochiensis (Gro)*, *Haemonchus contortus (Hco)*, *Heterodera glycines (Hgl)*, *Heterodera schachtii (Hsc)*, *Litomosoides sigmodontis (Lsi)*, *Meloidogyne arenaria (Mar)*, *Meloidogyne chitwoodi (Mch)*, *Meloidogyne hapla (Mha)*, *Meloidogyne javanica (Mja)*, *Meloidogyne incognita (Min)*, *Meloidogyne paranaensis (Mpa)*, *Necator americanus (Nam)*, *Nippostrongylus brasiliensis (Nbr)*, *Ostertagia ostertagi (Oos)*, *Onchocerca volvulus (Ovo)*, *Parascaris univalens (Pun)*, *Parastrongyloides trichosuri (Ptr)*, *Parelaphostrongylus tenuis (Pte)*, *Pristionchus pacificus (Ppa)*, *Pratylenchus penetrans (Ppe)*, *Pratylenchus vulnus (Pvu)*, *Strongyloides ratti (Sra)*, *Strongyloides stercoralis (Sst)*, *Toxocara canis (Tca)*, *Teladorsagia circumcincta (Tci)*, *Trichuris muris (Tmu)*, *Trichinella spiralis (Tsp)*, *Trichostrongylus vitrinus (Tvi)*, *Wuchereria bancrofti (Wbr)*, *Xiphinema index (Xin)*, *Zeldia punctata (Zpu)*.

**Figure 2:**
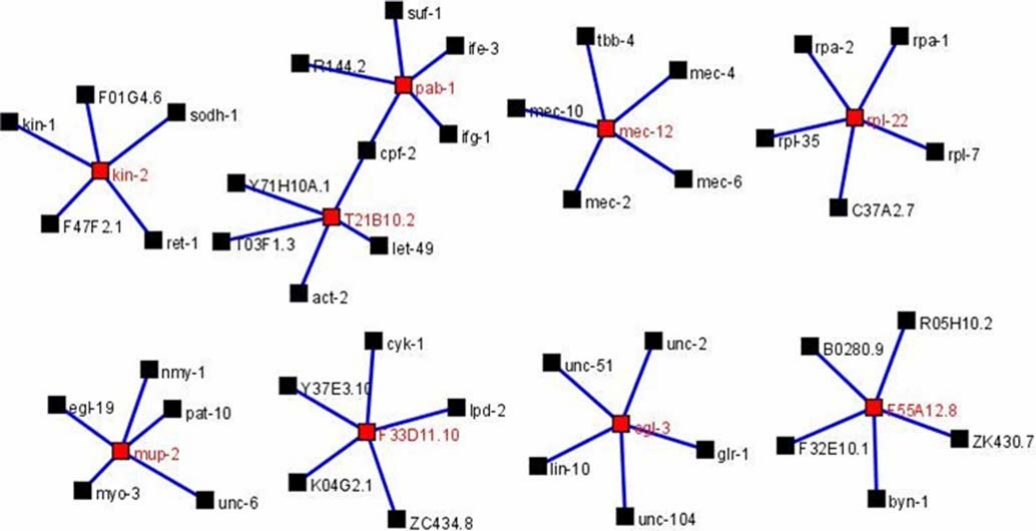
Probablistic gene interaction networking. The genetic interactions were predicted for a subset of nine *C. elegans* genes [*kin-2*, *pab-1*, T12B10.2, *mec-12*, *rpl-22*, *mup-2*, F33D11.10 ( = *enol-1*), *egl-3* and F55A12.8; in red] with homologues/orthologues in *Ascaris suum* which displayed significantly increased transcription in the infective third-stage larva (L3) compared with other developmental stages. Interactors are indicated in black.

Extending this analysis, a relatively complex genetic interaction network was predicted for the 17 *C. elegans* orthologues (i.e., with non-wild-type RNAi phenotypes) (see Table S2). Statistically highly significant interactions were predicted for nine of the *C. elegans* genes; the top five interactors are displayed in Fig. 2. The gene ontology categories for eight selected *C. elegans* genes (F33D11.10, F55A12.8, *kin-2*, *mec-12*, *mup-2*, *pab-1*, *rpl-22* and T21B10.2) included: embryonic development, egg hatching, larval development and/or growth. The other categories included: positive regulation of growth rate (F55A12.8, *kin-2*, *mup-2*, *pab-1*, *rpl-22* and T21B10.2) and gamete generation and locomotory behaviour (*kin-2*, *mup-2*, *pab-1* and F55A12.8, *kin-2*, *mup-2*, respectively). The *C. elegans* homologue *egl-3* was predicted to be involved in proteolysis (see www.wormbase.org). All nine *C. elegans* orthologues were predicted to interact directly with a total of 296 (range: 5–75) other genes and, in particular, a direct genetic interaction was predicted between *pab-1* and T21B10.2 (Fig. 2). The 296 interactors were associated with embryonic and larval development (n = 198; 66.9%), information storage and processing (n = 15; 5.1%), cellular processes and signalling (n = 45; 15.2%) and metabolism (n = 18; 6.1%); the precise function of some of the interactors (n = 20; 6.7%) is presently unknown (Table S2).

## Discussion

The present study investigated transcripts in infective L3s of *A. suum* using a genomic-bioinformatic platform. The focus was on comparisons with *C. elegans* homologues/orthologues, because the entire genome sequence of this nematode is known [9] and because there is a wealth of information on the localization and functionality of its molecules (www.wormbase.org; http://elegans.bcgsc.bc.ca/knockout.shtml). The functions of most genes in *C. elegans* have been assessed using RNAi (e.g., [14],[15],[17],[55],[56]) in the hermaphroditic stage, whereas there is a paucity of functional information available for *Ascaris* and other parasitic nematodes of animals [57],[58].

Following the microarray analysis of >2500 ESTs from the SSH library, 498 cDNAs inferred to be enriched in the L3, based on hybridization signal, were sequenced and subjected to comprehensive *in silico* analyses. Of the 91 clusters of molecules categorized, 50 (54.9%) had *C. elegans* homologues/orthologues with loss-of-function phenotypes could be mapped to key pathways. The statistically significant genetic interactions predicted for 9 of the 50 *C. elegans* orthologues [namely *egl-3*, F33D11.10, F55A12.8, *kin-2*, *mec-12*, *mup-2*, *pab-1*, *rpl-22* and T21B10.2 ( = *enol-1*)] and the interaction network included genes encoding kinases, alpha-tubulins, enolases, troponin and other named and unnamed proteins. Eight of these molecules (*enol-1*, *pab-1*, F33D11.10, *rpl-22*, F55A12.8 *mec-12*, *mup-2* and *kin-2*) have known or predicted roles in embryonic and larval growth and development, gamete generation, locomotory behaviour or other biological processes in *C. elegans* (see www.wormbase.org).

The enolase encoded by *enol-1* is predicted to play a role in glycolysis, gluconeogenesis, phenylalanine, tyrosine and tryptophan biosynthesis (cf. [59]). Since glucose is the main source for ATP production, the alteration in these key glycolytic enzymes may lead to cellular dysfunction, such as impaired ion-motive ATPase required to maintain potential gradients, operate pumps and maintain membrane lipid asymmetry [60]. Bioinformatic analysis for transmembrane helices (TMHMM) and peptide signal sequences (SignalP) predicted ENOL-1 to be a non-secreted protein localized to the cytoplasm (cf. Table 2). Nonetheless, enolases are often detected in the excretory/secretory (ES) products of parasitic helminths, including adult *A. suum* [61], and appear to play a role in the triggering of nitric oxide production by host cells. The *enol-1* orthologue of *C. elegans* has been predicted to interact specifically with the polyadenylate binding protein gene, *pab-1*, inferred to be involved in coordinated gene transcription and expression during normal larval development [16]. Poly(A)-binding proteins (PABPs) are recognized to be central to the regulation of mRNA translation and stability [62]. Present evidence suggests that the expression of PAB-1 is regulated by an oligo-pyrimidine tract in response to cell growth and relates to coordinated growth regulation in *C. elegans* [62]. Furthermore, gene silencing of *pab-1* and its selected interactors (see Fig. 2) leads to embryonic lethal (Emb), slow growth (Slo) and sterile progeny (Stp) phenotypes (see www.wormbase.org).

Another gene (F33D11.10; EST code 4F10; see Table 2) which encodes an ATP-dependent RNA helicase and is associated with embryonic lethal (Emb) and larval lethal (Lvl) RNAi phenotypes, was shown to be highly transcribed in infective L3s of *A. suum*. Helicases are involved in a variety of RNA metabolic processes, including translation initiation, pre-mRNA splicing, pre-rRNA processing, rRNA maturation and RNA degradation [63], and are crucial for life cycle progression, sex determination and early embryogenesis in *C. elegans* [60]. The high transcription levels of a homologue/orthologue in the L3 of *A. suum* might suggest a similar role in this ascaridoid. Similarly, the coordination of the expression of a large number of genes is required for normal growth and cell proliferation during larval development. The high transcription level for the ribosomal protein gene homologue *rpl-22* (large subunit family member; EST code 26G12, see Table 2) in the infective L3 of *A. suum* compared with other developmental stages is likely to reflect the substantial rate of cell growth in this stage [64].

The gene (F55A12.8, EST code 4G11; see Table 2) encoding an acetyl-transferase with a putative ATPase domain, shown to be enriched in the L3 of *A. suum*, was predicted to interact with 75 other genes all involved in energy production and/or RNA processing (see Table S2). Several molecules involved in ATP synthesis and mitochondrial pathways (e.g., cytochrome oxidase *c* subunits 1, 2 and 3, ADP/ATP translocases, NADH dehydrogenases, ATPases and ATP synthetases) have been reported previously to be highly represented in the L3 stage of *Anisakis simplex* [65], thus supporting the proposal that substantial energy is required for larval development as well as the transition from the free-living to the parasitic stage and the invasion of the host. There is also likely to be a substantial energy requirement for muscle contraction linked to larval motility in *A. suum*, as the L3s penetrate the caecal wall in the porcine host, before undergoing hepato-pulmonary migration [35]. Accordingly, genes encoding a specialized tubulin expressed in mechanoreceptors (*mec-12*, EST code 13E09) and a troponin (*mup-2*, EST code 01G03; see Table 2), both predicted to interact with a total number of 32 tubulin- and myosin-encoding genes, also supported a link to extensive muscle contraction and motility in *A. suum* L3s. Also, neuroactive peptides are required to regulate the responsiveness of nematode larvae to mechanical stimuli [66]. A homologue encoded by *egl-3* was shown to be highly transcribed in the L3 of *A. suum*; EGL-3 is predicted to be a pro-hormone convertase involved in the maturation of neuropeptides and could be associated with mechano-sensory responses and touch sensitivity linked to the host invasion.

A regulatory subunit of a cAMP-dependent protein kinase (*kin-2*, EST code 22H01; see Table 2) was predicted to interact with 72 other genes all involved in diverse cellular processes, such as nuclear trafficking, and DNA replication and repair (see Table S2). Based on gene ontology terms, *kin-2* is implicated in gamete generation, growth, larval development, post-embryonic body morphogenesis, signal transduction and/or protein amino acid phosphorylation (see Table S2). Gene silencing of *kin-2* in *C. elegans* leads to phenotypes, such as larval lethal (Lvl), larval arrest (Lva), body morphology defect (Bmd), dumpy (Dpy), uncoordinated (Unc) and sterile progeny (Stp) (www.wormbase.org), suggesting that its homologue in *A. suum* is central to larval maturation. The KOBAS analysis predicted the protein KIN-2 to be involved in the insulin-signaling pathway, previously implicated in controlling the exit from dauer in *C. elegans* and the activation of L3s of the canine hookworm, *Ancylostoma caninum*, following exsheathment [67]. In a recent study, Brand and Hawdon [68] were able to inhibit (with a phosphoinositide-3-OH-kinase inhibitor) the activation of infective L3s of both of the hookworms *Ancylostoma caninum* and *Ancylostoma ceylanicum* via the insulin signaling pathway, thus lending some credence to the hypothesis that this pathway plays an critical role in regulating the transition from the free-living to the parasitic stage [68]. Recently, it has been proposed that transcriptional and feeding responses to serum-stimulation in *Ancylostoma caninum* are regulated by parallel systems, with the insulin signaling pathway playing a significant role in the ‘resumption of feeding’ in activated larvae [69].

Protein kinases are also likely to be involved in pathways linked to sexual maturation in developing larvae. As already proposed for adult stages of *H. contortus* [45], the protein kinase gene *cdk-1* is predicted to play a pivotal role in the germline, oogenesis and spermiogenesis pathways of this parasitic nematode. Other protein kinases, such as PEPCK, and phosphatases, were shown herein to be transcribed at high levels in the L3 stage compared with other developmental stages of *A. suum* (see Table 2), which is in accordance to findings reported recently for *Anisakis simplex* [65]. Due to their major regulatory effects in eukaryotic signaling events and regulatory and sensory functions, protein kinases have been considered interesting targets for anti-parasitic drugs [70].

In conclusion, this study has given some interesting insights into early molecular processes in the L3 of *A. suum*. Approximately 60% of the transcripts enriched in the L3 stage of *A. suum* have homologues/orthologues in *C. elegans*. The bioinformatic analyses of selected molecules suggest that a complex genetic network regulates or controls larval growth and development in *A. suum* L3s, and some of these might be involved in or regulate the switch from the free-living to the parasitic stage. Some caution is warranted in drawing conclusions regarding molecular mechanisms regulating the transition to parasitism in parasitic nematodes from information on *C. elegans*, as latter is a free-living nematode. Also, while the method of data integration is essential for the reliable prediction of genetic interactions, it might limit the capacity of the approach somewhat to infer nematode-specific interactions. As additional datasets of genes and gene functions become available for various parasitic nematodes, more informed inferences can be made regarding the functions of nematode-specific genes, particularly those involved in the transition to parasitism. The imminent genome sequence of *A. suum* (http://www.sanger.ac.uk/Projects/Helminths/) should all assist in this endeavour. Also, functional analysis of selected molecules representing selected ESTs identified herein, utilizing gene silencing approaches established recently [33],[34], could provide some insights into developmental processes in *Ascaris* and related ascaridoid nematodes and provide avenues for the development of novel approaches for their control.

## Supporting Information

Table S1 Numbers and percentages of annotated clusters of molecules enriched in the infective third-stage larva (L3) of *Ascaris suum*, classified according to gene ontology (GO) categories ‘biological process’, ‘cellular component’ and ‘molecular function’.
(0.03 MB XLS)

Table S2 Genes (n = 296) predicted to interact with each of nine selected *C. elegans* genes [kin-2, pab-1, T12B10.2, mec-12, rpl-22, mup-2, F33D11.10 ( = enol-1), egl-3 and F55A12.8], listed according decreasing cut-off score. Gene ontology (GO) terms and known RNAi phenotypes for each interacting gene are also listed.
(0.13 MB XLS)
